# Metabolic Effects of Clenbuterol and Salbutamol on Pork Meat Studied Using Internal Extractive Electrospray Ionization Mass Spectrometry

**DOI:** 10.1038/s41598-017-05496-6

**Published:** 2017-07-11

**Authors:** Haiyan Lu, Hua Zhang, Tenggao Zhu, Yipo Xiao, Shaoxian Xie, Haiwei Gu, Meng Cui, Liping Luo

**Affiliations:** 1 Jiangxi Key Laboratory for Mass Spectrometry and Instrumentation, East China University of Technology, Nanchang, 330013 P. R. China; 2 Sports Science Institute of Jiangxi Province, Nanchang, 333006 P. R. China; 3 State Key Laboratory of Food Science and Technology, School of Life Sciences, Nanchang University, Nanchang, 330047 P. R. China

## Abstract

Direct mass spectrometry analysis of metabolic effects of clenbuterol and salbutamol on pork quality at the molecular level is incredibly beneficial for food regulations, public health and the development of new anti-obesity drugs. With internal extractive electrospray ionization mass spectrometry (iEESI-MS), nutrients including creatine, amino acids, L-carnitine, vitamin B_6_, carnosine and phosphatidylcholines in pork tissue were identified, without sample pretreatment, using collision-induced dissociation (CID) experiments and by comparison with authentic compounds. Furthermore, normal pork samples were clearly differentiated from pork samples with clenbuterol and salbutamol via principal component analysis (PCA). Correlation analysis performed on the spectral data revealed that the above-mentioned nutrients strongly correlated with pork quality, and the absolute intensity of phosphatidylcholines in normal pork was much higher than pork contaminated by clenbuterol and salbutamol. Our findings suggested that clenbuterol and salbutamol may render effects on the activity of carnitine acyltransferase I, hence the process that L-carnitine transports long-chain fatty acids into mitochondria and the formation of phosphatidylcholines might be affected. However, the underlying metabolic mechanisms of clenbuterol and salbutamol on carnitine acyltransferase I requires more comprehensive studies in future work.

## Introduction

Pork has high nutritional value and is one of the most commonly consumed meat﻿s in the public. However, clenbuterol contamination scandal^1, 2^ has raised worldwide concerns on meat and meat products quality in recent decades^3^. β-agonists, a class of phenylethanolamine compounds similar in chemical structures, are widely used for the treatment of respiratory diseases in clinical applications^4, 5^. It has been reported that β-agonists promote animal growth rates and increase muscle leanness by inducing redistribution of fat in the muscle tissues of mammals^6–8^. Driven by reaping high profits, β-agonists are often illegally fed to animals. However, the residues of these chemicals pose potential hazards to human health, with serious symptoms such as muscular tremors, cardiac palpitations, headaches, and muscular pain, even cause life threatening cardiovascular and central nervous disease by increasing liver mass, changing enzyme activity, and alternating blood cell proportions and hormone levels^9, 10^. Furthermore, β-agonists could stimulate glycogenolysis in muscle tissues, which limits normal postmortem acidification and promotes the development of dark, firm, dry (DFD) meat.

Currently, many researches were mainly limited to the development of simple, rapid, and sensitive methods for determination β-agonists residues in various biological samples, and evaluations of β-agonists on animal growth performance. The data on the impact of β-agonists on glucose production, insulin release and lipolysis were presented^11^. Based on chemical properties (moisture, ash, fat and protein) and physical properties (pH, tenderness, cooking shrinkage and welted holding capacity), the effects of clenbuterol on meat quality of growing male pig were analyzed^12^. Besides, researches about the effects of β-agonists have been done on beef and sheep, and the effects of clenbuterol and salbutamol on tissue Rubidium uptake *in vivo* ^13^ and meat quality in veal calves^14^ were reported. Nevertheless, few efforts have been made to explore the metabolic effects of β-agonists, especially the widely used clenbuterol and salbutamol, on meat tissue of mammals such as pork.

To investigate the metabolic effects of clenbuterol and salbutamol on meat quality of mammals (e.g., pork), it is desirable to obtain the metabolic profile of pork tissue by keeping the tissue samples at the native status to the maximal degree. In past decades, analytical methods such as liquid chromatography-mass spectrometry (LC-MS)^15^, enzyme-linked immunosorbent assay (ELISA)^16^, gas chromatography-mass spectrometry (GC-MS)^17^, surface-enhanced raman scattering (SERS)^18^ and polymer monolith microextraction combined with electrospray ionization quadrupole time-of-flight mass spectrometry (PMEM/ESI-Q-TOF-MS)^19^ have been developed for the analysis of trace β-agonists residues in various food matrices. So far there have been no reports available for direct detection of β-agonists embedded in meat tissue that do not include time-consuming procedures such as grinding, extraction and centrifugation. Thus, novel analytical protocols are demanded to record the molecular profiling of the metabolites in the native state of pork tissue.

Here, internal extractive electrospray ionization mass spectrometry (iEESI-MS)^20–22^, which combines solvent extraction of chemical components inside a bulk tissue sample with *in situ* electrospray ionization mass spectrometry without either mashing/grinding the sample or matrix clean-up, was used for direct analysis of metabolic effects of clenbuterol and salbutamol on pork quality at the molecular level. Moreover, correlation analysis performed on the spectral data revealed that some nutrients strongly correlated with pork quality, and the absolute intensities of phosphatidylcholines in normal pork samples were much higher than in the samples contaminated by clenbuterol and salbutamol. These findings suggested that clenbuterol and salbutamol may render effects on the activity of carnitine acyltransferase I, hence the formation of phosphatidylcholines might be affected. However, the underlying metabolic mechanisms of clenbuterol and salbutamol on carnitine acyltransferase I require more comprehensive studies in future work.

## Results

### Direct iEESI-MS analysis of normal pork and pork with clenbuterol and salbutamol

**Figure 1:**
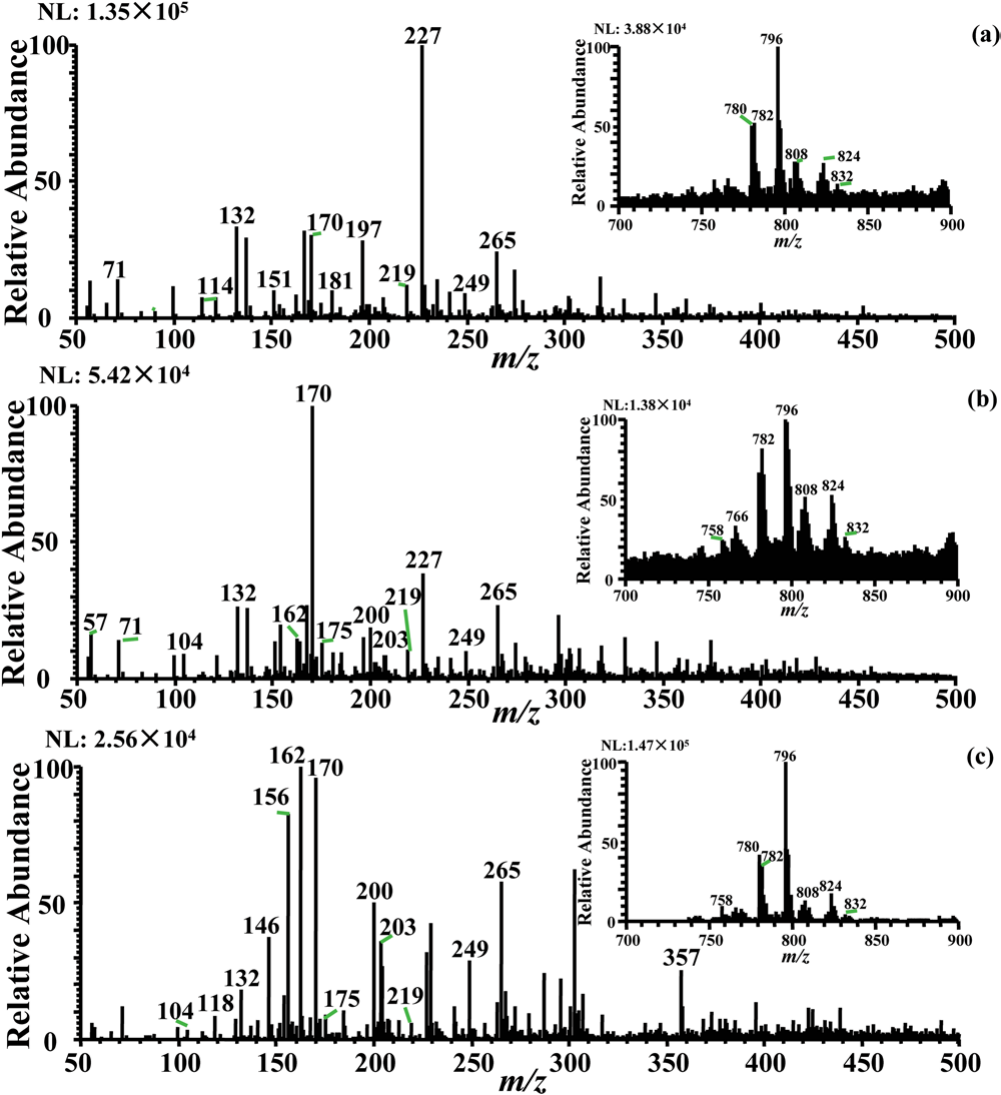
iEESI-MS analysis of three types of pork samples. (**a**) Pork with clenbuterol; (**b**) pork with salbutamol; (**c**) normal pork.

Figure 1 shows the characteristic chemical fingerprints of pork with clenbuterol (Fig. 1a), pork with salbutamol (Fig. 1b) and normal pork (Fig. 1c) in the low mass range (*m*/*z* 50–500) and middle mass range (*m*/*z* 700–900), which were obtained by iEESI-MS under the positive ion detection mode using CH_3_OH:CH_3_COOH = 98:2 (v/v) as extraction solvents. Due to the *in situ* extraction/ionization of pork samples, ionic inorganic species including Na^+^ and K^+^ acted as cationic reagents during the ionization process. A large amount of chemical components were successfully detected from pork tissues and identified based on the CID experiments, the NIST/EPI/NIH Mass Spectral Library, authentic compounds (Figure S1) and literatures data. The majority of dominant peaks in both normal pork sample and pork contaminated by clenbuterol and salbutamol at mass range of *m*/*z* 50–500 were assigned as amino acids^20, 23, 24^ (e.g., *m*/*z* 118 [Valine+H]^+^, *m*/*z* 147 [Lysine+H]^+^, *m*/*z* 156 [Histidine+H]^+^, *m*/*z* 175 [Arginine+H]^+^), sugars (*m*/*z* 219 [Glucose+K]^+^), alkaloids (*m*/*z* 104 [Choline]^+^), polyamine (*m*/*z* 203 [Spermine+H]^+^), and *m*/*z* 132 [Creatine+H]^+^^25^, *m*/*z* 162 [L-carnitine+H]^+^, *m*/*z* 170 [Vitamin B_6_+H]^+^, *m*/*z* 227 [Carnosine+H]^+^^26^. The main peaks at mass range of *m*/*z* 700–900 were identified as phosphatidylcholines (PCs) including *m*/*z* 808 [PC (36:2)+Na]^+^, *m*/*z* 824 [PC (36:2)+K]^+^, *m*/*z* 782 [PC (36:4)+H]^+^, *m*/*z* 780 [PC (34:2)+Na]^+^, *m*/*z* 796 [PC (34:2)+K]^+^ and *m*/*z* 832 [PC (38:4)+Na]^+^^27–29^, etc. Identification of part of the nutrients in pork samples was shown in Table 1.

**Table 1:** Identification of part of the nutrients in pork samples.

Compounds	Molecular formula	*m*/*z*	Ion formation	MS/MS fragments	References
Valine	C_5_H_11_NO_2_	118	[M+H]^+^	101, 72	20, 23, 24, authentic compounds
Creatine	C_4_H_9_N_3_O_2_	132	[M+H]^+^	114, 90, 86	25, authentic compounds
Lysine	C_6_H_14_N_2_O_2_	147	[M+H]^+^	130, 84	20, 23, 24, authentic compounds
Histidine	C_6_H_9_N_3_O_2_	156	[M+H]^+^	112, 110, 74	23, 24, authentic compounds
Arginine	C_6_H_14_N_4_O_2_	175	[M+H]^+^	158, 157, 130, 116, 70, 60	20, 23, 24, authentic compounds
L-carnitine	C_7_H_15_NO_3_	162	[M+H]^+^	103, 102, 85, 60	authentic compounds, NIST/EPI/NIH Mass Spectral Library
Vitamin B_6_	C_8_H_11_NO_3_	170	[M+H]^+^	152, 134	authentic compounds, NIST/EPI/NIH Mass Spectral Library
Spermine	C_10_H_26_N_4_	203	[M+H]^+^	129, 112	authentic compounds, NIST/EPI/NIH Mass Spectral Library
Carnosine	C_9_H_14_N_4_O_3_	227	[M+H]^+^	210, 192, 164, 110	26, NIST/EPI/NIH Mass Spectral Library
PC(34:2)	C_42_H_80_NO_8_P	780	[M+Na]^+^	721, 597	28
PC(36:4)	C_44_H_81_NO_8_P	782	[M+H]^+^	723, 599	28, 29
PC(34:2)	C_42_H_80_NO_8_P	796	[M+K]^+^	737, 613	28
PC(36:2)	C_44_H_84_NO_8_P	808	[M+Na]^+^	749, 625	27, 28
PC(36:2)	C_44_H_84_NO_8_P	824	[M+K]^+^	766, 642	27, 28
PC(38:4)	C_46_H_84_NO_8_P	832	[M+Na]^+^	774, 649	27, 28

It should be noted that significant differences were observed between the mass spectra of three types of pork samples. For example, the peak at *m*/*z* 227 [Carnosine+H]^+^ dominated in the spectra of pork with clenbuterol (Fig. 1a), while [Vitamin B_6_+H]^+^ at *m*/*z* 170 dominated in the spectra of pork with salbutamol (Fig. 1b). The relative abundance of [Histidine+H]^+^ at *m*/*z* 156 and [L-carnitine+H]^+^ at *m*/*z* 162 in normal pork was significantly higher than the pork with clenbuterol and salbutamol, whereas the relative abundance of [Creatine+H]^+^ at *m*/*z* 132 in the pork with clenbuterol and salbutamol was slightly higher than normal pork. The absolute signal intensities of phosphatidylcholines at *m*/*z* 700–900 in normal pork were, on the average, 1 order of magnitude higher than it in the pork containing clenbuterol and salbutamol.

### Differentiation of normal pork and pork with clenbuterol and salbutamol

**Figure 2:**
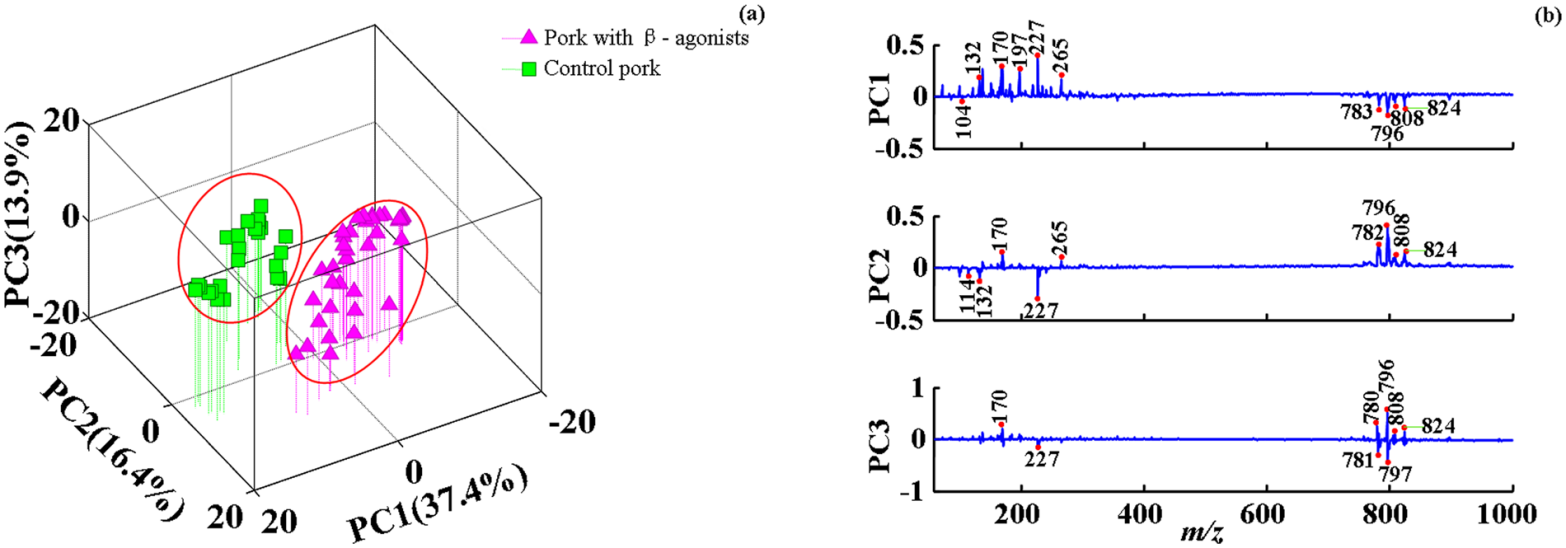
Differentiation of normal pork and pork with β-agonists residues. (**a**) 3D-PCA score plots; (**b**) PCA loading plots.

3D-PCA score plots further revealed the meat quality differences between normal pork and pork contaminated by clenbuterol and salbutamol. As shown in Fig. 2a, normal pork samples were successfully differentiated from pork samples with clenbuterol and salbutamol based on the first three principal components via PCA. And it could be seen from PCA loading plots (Fig. 2b) that nearly all signal peaks of phosphatidylcholines such as *m*/*z* 782, 796, 808, and 824 in mass range of 700–900 as well as part of small-molecule compounds including *m*/*z* 132, 170, 227 and 265 are the main contributors to the differentiation.

### Correlation analysis of small-molecule compounds and phosphatidylcholines in three types of pork samples

**Figure 3:**
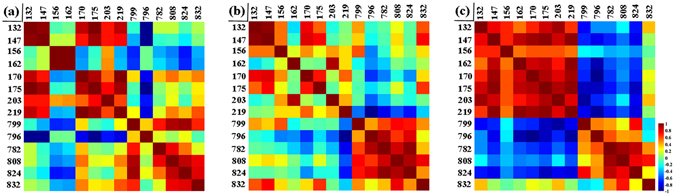
Correlation among the small-molecule compounds with phosphatidylcholines in three types of pork samples. Selecting higher relative abundances of ions at *m*/*z* 132 [Creatine+H]^+^, *m*/*z* 147 [Lysine+H]^+^, *m*/*z* 156 [Histidine+H]^+^, *m*/*z* 162 [L-carnitine+H]^+^, *m*/*z* 170 [Vitamin B_6_+H]^+^, *m*/*z* 175 [Arginine+H]^+^, *m*/*z* 203 [Spermine+H]^+^, *m*/*z* 219 [Glucose+K]^+^, *m*/*z* 799 [PC (36:4)+K]^+^, *m*/*z* 796 [PC (34:2)+K]^+^, *m*/*z* 782 PC (36:4)+H]^+^, *m*/*z* 808 [PC (36:2)+Na]^+^, *m*/*z* 824 [PC (36:2)+K]^+^ and *m*/*z* 832 [PC (38:4)+Na]^+^ in pork with clenbuterol, pork with salbutamol, and normal pork were performed correlation analysis, respectively. From green to red (the value range from 0 to 1) indicated that the positive correlation gradually increased; from green to blue (the value range from 0 to −1) indicated that the negative correlation gradually increased. (**a**) Pork with clenbuterol; (**b**) pork with salbutamol; (**c**) normal pork.

To further probe the mechanism of metabolic effect of clenbuterol and salbutamol on pork quality, 14 kinds of higher relative abundances ions including *m*/*z* 132 [Creatine+H]^+^, *m*/*z* 147 [Lysine+H]^+^, *m*/*z* 156 [Histidine+H]^+^, *m*/*z* 162 [L-carnitine+H]^+^, *m*/*z* 170 [Vitamin B_6_+H]^+^, *m*/*z* 175 [Arginine+H]^+^, *m*/*z* 203 [Spermine+H]^+^, *m*/*z* 219 [Glucose+K]^+^, *m*/*z* 799 [PC (36:4)+K]^+^, *m*/*z* 796 [PC (34:2)+K]^+^, *m*/*z* 782 PC (36:4)+H]^+^, *m*/*z* 808 [PC (36:2)+Na]^+^, *m*/*z* 824 [PC (36:2)+K]^+^ and *m*/*z* 832 [PC (38:4)+Na]^+^ in pork with clenbuterol (Fig. 3a), pork with salbutamol (Fig. 3b) and normal pork (Fig. 3c) were selected for correlation analysis. As shown in Fig. 3, [Creatine+H]^+^ at *m*/*z* 132 was strongly and positively correlated with [Lysine+H]^+^ at *m*/*z* 147, [Vitamin B_6_+H]^+^ at *m*/*z* 170 and [Arginine+H]^+^ at *m*/*z* 175. Interestingly, [Creatine+H]^+^ at *m*/*z* 132 had a positive correlation with [Spermine+H]^+^ at *m*/*z* 203 and [Glucose+K]^+^ at *m*/*z* 219 in pork with clenbuterol and normal pork, whereas it had a weak negative correlation with [Spermine+H]^+^ at *m*/*z* 203 and [Glucose+K]^+^ at *m*/*z* 219 in pork with salbutamol. Moreover, it could be evidently seen from normal pork (Fig. 3c) that nearly all these small-molecule compounds including *m*/*z* 132, 147, 156, 162, 170, 175, 203 and 219 had negative correlations with most of the phosphatidylcholines and positive correlations with each of them, whereas phosphatidylcholines showed positive correlations with each of them. Overall, there was no regular correlation relationship of above-mentioned compounds in pork contaminated by clenbuterol (Fig. 3a) and salbutamol (Fig. 3b) compared with normal pork, and the correlation relationship of different compounds in normal pork were much more regular than those in pork containing clenbuterol and salbutamol.

Lysine, as a precursor of L-carnitine, is absorbed by the gut, and part of it synthesizes N-trimethyllysine in a reaction catalyzed by aminotransferase, ultimately, N-trimethyllysine is catalyzed by a series of enzymes to form L-carnitine. L-carnitine is a quaternary ammonium compound, which is involved in the energy metabolism and yielded from lysine and methionine or taken up with diet. As an essential factor in fatty acid metabolism in mammals, its most important known metabolic function is to transport fatty acids into the mitochondria compartment^30^. As their correlation associations shown in Fig. 3, [Lysine+H]^+^ at *m*/*z* 147 was positively correlated with [L-carnitine+H]^+^ at *m*/*z* 162, and both of them were negatively correlated with phosphatidylcholines including *m*/*z* 799, 796, 782, 808, 824 in normal pork (Fig. 3c). However, [Lysine+H]^+^ at *m*/*z* 147 was negatively correlated with [L-carnitine+H]^+^ at *m*/*z* 162 in pork with cenbuterol (Fig. 3a) and salbutamol (Fig. 3b), and both lysine and L-carnitine had no regular correlation with phosphatidylcholines in pork with clenbuterol (Fig. 3a) and salbutamol (Fig. 3b).

### Association of the relative abundance ratios of phosphatidylcholines/L-carnitine in three types of pork samples

**Figure 4:**
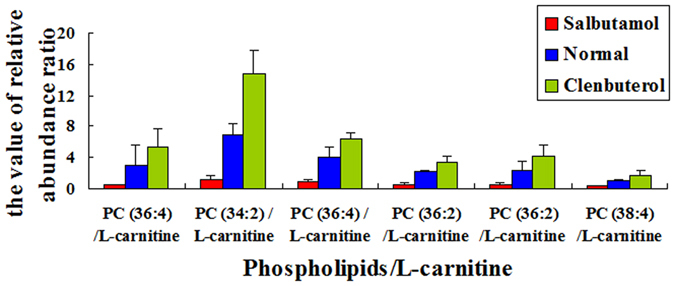
The relative abundance ratios of phosphatidylcholines/L-Carnitine in three types of pork samples. The ratios of phosphatidylcholines/L-Carnitine are [PC (36:4)+K]^+^/L-carnitine, [PC (34:2)+K]^+^/L-carnitine, PC (36:4)+H]^+^/L-carnitine, [PC (36:2)+Na]^+^/L-carnitine, [PC (36:2)+K]^+^/L-carnitine and [PC (38:4)+Na]^+^/L-carnitine. (Red bar: pork with salbutamol; blue bar: normal pork; green bar: pork with clenbuterol.)

L-carnitine plays an important physiological role in lipid metabolism, especially in the transport of long-chain fatty acids into inner mitochondrial, and fatty acids play a key role in formation of phosphatidylcholines. Here, the relative abundance ratios of phosphatidylcholines/L-carnitine (e.g., [PC (36:4)+K]^+^/L-carnitine, [PC (34:2)+K]^+^/L-carnitine, [PC (36:4)+H]^+^/L-carnitine, [PC (36:2)+Na]^+^/L-carnitine, [PC (36:2)+K]^+^/L-carnitine and [PC (38:4)+Na]^+^/L-carnitine) in pork with clenbuterol, pork with salbutamol, and normal pork were investigated, respectively. As shown in Fig. 4, it is worth noting that the relative abundance ratios of above-mentioned phosphatidylcholines/L-carnitine in pork with clenbuterol (Fig. 4 green bar) were significantly higher than those in the normal pork (Fig. 4 blue bar) and pork with salbutamol (Fig. 4 red bar).

## Discussion

With no/minimal sample pretreatment, ambient ionization techniques such as desorption electrospray ionization mass spectrometry (DESI-MS)^31^ and direct analysis in real time mass spectrometry (DART-MS)^32^ have been applied to the direct detection of analytes in complex matrices. However, these techniques mainly conduct analysis on the sample surface that may not exactly reflect the chemical content within the bulk volume. Based on the modification of electrospray ionization mass spectrometry (ESI-MS), probe electrospray ionization mass spectrometry (PESI-MS)^33^, paper spray ionization mass spectrometry^27^ and tissue spray ionization mass spectrometry (TSI-MS)^34–37^ have the been developed to analyze biofluids and tissue samples, which further extended the application scope of ambient MS.

Both iEESI-MS and TSI-MS are featured by rapid and throughput analysis, without affecting sample integrity and no auxiliary gas. In TSI-MS analysis, analyte molecules can be directly sprayed and ionized from the solvent-wetted tissues when the application of a high voltage^36^. While in iEESI-MS, extraction solution biased with high voltage is continuously infused into bulk tissue sample through the parallelly inserted capillary for the extraction of “internal” chemicals from a bulk tissue sample. Hence stable and continuous electrospray plume is produced in front of MS inlet. Compared with TSI-MS, the unique property of iEESI-MS is that allowed direct analysis of much more interior chemicals from bulk samples with molecular specificity. In addition, previous literatures reported that iEESI-MS has been successfully used for direct profiling of various biological tissue samples, with satisfactory qualitative and quantitative performance. In this regard, the iEESI-MS is a suitable technique for profiling of pork tissue samples, and visualization the metabolic effects of clenbuterol and salbutamol on pork meat quality at molecular level.

To investigate the metabolic effects of clenbuterol and salbutamol on meat quality of mammals (e.g., pork), it is desirable to obtain the metabolic profile of pork tissue by keeping the tissue samples at the native status to the maximal degree. In this study, metabolic effects of clenbuterol and salbutamol on pork quality were directly evaluated by iEESI-MS at the molecular level. Our results showed that absolute intensity of phosphatidylcholines in normal pork was higher than pork contaminated by clenbuterol and salbutamol, this might be largely attributed to the effect of β-agonists on lipids metabolism, possibly affecting lipolysis or lipogenesis, and a reduction in total fat-cell numbers^38^. According to the correlation relationship of L-carnitine and phosphatidylcholines in three types of pork samples showed in Fig. 3, it could be clearly observed that the correlation relationship of L-carnitine and phosphatidylcholines in three types of pork samples was significantly different. This could be proposed that clenbuterol and salbutamol may render effects on the activity of carnitine acyltransferase I, which regulated biosynthesis reaction from long-chain acyl-CoA and L-carnitine to acylcarntine and CoA, therefore, the process of long chain acyl-CoA into the mitochondria compartment and the formation of phosphatidylcholines might be affected. Besides, as shown in Fig. 4, associations of the relative abundance ratios of phosphatidylcholines/L-carnitine in three types of pork samples also revealed that specific β-agonists might have specific effects on the change of components in pork due to differences in chemical and pharmacokinetic properties^39^.

For example, previous studies on farm animals indicated that the highest accumulation of clenbuterol and salbutamol is to be expected in pigmented tissues, such as eye and hair, which can be explained by their high melanin binding affinity, liposolubility, and pH gradient between blood and hair^40^. Irrespective of the applied dose, salbutamol residue concentration is generally lower in quantity compared with clenbuterol residue concentration^41^. Though urine is the main metabolic pathway of β-agonists in all species including humans, the pigs that were administered salbutamol had softer fat that tended to separate from the underlying lean tissue, and salbutamol itself did not exacerbate the loss of fat firmness related to an increase of leanness^42^. However, clenbuterol takes part into the biotransformation including both Phase I and Phase II metabolism, yielding various metabolites detected in the urine of different animals^43^.

Our findings proposed that clenbuterol and salbutamol may render effects on the activity of carnitine acyltransferase I, and the process that L-carnitine transports long-chain fatty acids into mitochondria and the formation of phosphatidylcholines might be affected. Although underlying mechanisms of clenbuterol and salbutamol on carnitine acyltransferase I are not completely known, and no available literature presented that carnitine acyltransferase I was one of critical target of β-agonists, while protein kinase A, adenylate cyclase and citrate synthase have previously been reported as the target of clenbuterol^44, 45^. Specifically, L-carnitine could affect the lipids metabolism, studies reported that increases in L-carnitine level in the liver and serum may accelerate the transport of acyl-CoA dehydrogenase to mitochondria, enhance the oxidation of fatty acids and decrease liver and serum triglyceride (TG)^46^. And L-carnitine could reduce the deposition of fat in animal bodies by decreasing the formation of fat^47^. These might herald that carnitine acyltransferase I and acyl-CoA synthase related to fatty acids metabolism were of special interest for further study.

## Methods

### Pork samples

All animal care and experimentations conducted were approved by the Animal Ethics Committee of Nanchang University in accordance with the guidelines of Gan (Dong) 2012021. Eighteen two month-old male Gandong black pigs were cultured in the identical environment and administrated with the same diet in the same way. All of these pigs were castrated during lactation, and the weight of these pigs was in the same level. They were mainly fed with coarse feed including bran and vegetables three times a day, once in the morning, afternoon and evening, respectively. Except for the experimental group where the diet was spiked with clenbuterol (daily dose of 5 mg/kg) or salbutamol (daily dose of 5 µg/kg) for 90 days before the meat was properly harvested. The pork samples analyzed were all lean tissues on the same sites of the left shoulders of different pigs, which were provided by Sports Science Institute of Jiangxi Province. All collected pork samples were stored at around −80 °C in an ultra-low refrigerator, but were allowed to come to room temperature before performing iEESI-MS analysis.

In this study, one pig provided one sample, a total of 18 pork samples from 18 pigs (control group including 9 normal pork samples, experimental group including 2 pork samples with clenbuterol and 7 pork samples with salbutamol) were experimentally analyzed using iEESI-MS, one sample analyzed 6 times. To standardize the sample operation, the tissue samples from identical sections were cut into a fixed shape with the thickness of 3 mm and the side length of 10 mm for each analysis.

### Chemicals

Both methanol and acetic acid were HLPC grade and bought from ROE Scientfic Inc. (Newark, U.S.A). All amino acids authentic compounds such as valine, histidine, arginine and lysine were purchased from Sinopharm Chemical Reagent Co. Ltd. (Shanghai, China). Creatine, L-carnitine (BR) and vitamin B_6_ (BR) were purchased from Shanghai Source Leaf Biological Technlogy Co., Ltd (Shanghai, China) with 98%, 98% and 99% purity, respectively. The deionized water used for experiments was provided by a Milli-Q water purification system (Billerica, USA). All standard solutions were prepared in deionized water and then used for ESI-MS analysis without further treatment.

### Instrumentation and working conditions

The iEESI-MS experiments were carried out under positive ion detection mode using a homemade iEESI ion source coupled with a LTQ mass spectrometer (Thermo Scientific, San Jose, U.S.A), which was equipped with Xcalibur software for instrument control and data processing. Schematic illustration of iEESI-MS for direct characterization of bulk tissue samples was as described in previous literatures^20–22^. The detailed iEESI-MS experiments operation conditions were as follows^48^: the voltage used for spray ionization was 5.5 kV. The temperature of the MS capillary inlet was set at 150 °C. The tube lens voltage was set at 100 V and the capillary voltage was 10 V. CH_3_OH:CH_3_COOH = 98:2 (v/v) was used as extraction solvent for all the following iEESI-MS experiments, the flow rate of extraction solvent was kept at 0.5–1 μL/min.

The ESI-MS experiments were also performed on a LTQ mass spectrometer (Thermo Scientific, San Jose, U.S.A) under positive ion detection mode. Standard solution charged with 4.0 kV was injected at a flow rate of 5 µL/min by a syringe (250 µL, Hamilton, GR, Switerland) with a syringe pump (Harvard, MA, USA) for ESI-MS analysis. Nitrogen gas (Jiangxi Guoteng Gas Co., Ltd., China) pressure was 0.8 MPa.

For CID experiments, the MS^n^ spectrum for ion detection used a maximum injection time of 100 ms, the precursor ion isolation window of 1.5 Da and normalized collision energy of 10–30% were carried out on the characteristic ions for molecular structure confirmation. Other parameters were set as default values of the instrument, no further optimization was performed.

### iEESI-MS methods validation

Previous literature reported that the quantitative propriety of iEESI-MS was experimentally investigated^22^. Bulk sample of pork meat spiked with different concentration of salbutamol were analyzed by iEESI-MS/MS, the experiment result demonstrated that the limit of detection (LOD, S/N = 5) of this method was 0.0399 µg/L, and the relative standard deviation (RSD) was in the range of 6–15% (7 pork samples). In order to obtain better results, some parameters such as the volume and shape of pork samples, the distance between ESI capillary tip and sample edges as well as the distance between sample edges and inlet of mass spectrometry should be kept fixed.

### PCA analysis

PCA was used to recognize the pattern based on the mass spectral raw data using the Matlab (version 7.8.0, Mathworks, Inc., Natick, MA). The full-scan mass spectral (MS^1^) data was exported into Microsoft Excel and arranged using the *m*/*z* values as independent variables and the signal intensity of MS^1^ as dependent variables. All of the mass spectral data expressed in signal intensity were further loaded into the Matlab software for PCA analysis. The principal components for output were automatically determined by the “Princomp” function in the “Matlab Toolbox”. Hence the score plots and loading plots of the first three principal components of normal pork samples and pork containing β-agonists were visually presented. When exporting mass spectra data into Microsoft Excel, a total of 108 data points form 18 pork samples (six data points per sample on average) were interpreted in the score graphs of the PCA.

### Correlation analysis

Person correlation analysis using SPSS 18.0 (SPSS Inc., Chicago, IL, USA) was carried out among the relative abundance of 14 kinds of nutrients in pork with clenbuterol, pork with salbutamol, and normal pork. The relative abundance of nutrients with p value of less than 0.05 was considered to be statistically significant. The result of person correlation analysis was exported into Microsoft Excel for further picture presentation by program in Matlab.
